# Cataract Surgery in Eyes with Previous Glaucoma Surgery: Pearls and Pitfalls

**DOI:** 10.5005/jp-journals-10008-1145

**Published:** 2013-09-06

**Authors:** Tanuj Dada, Shibal Bhartiya, Nafees Begum Baig

**Affiliations:** Professor, Department of Ophthalmology, Dr RP Centre for Ophthalmic Sciences, All India Institute of Medical Sciences, New Delhi, India; Consultant, Department of Ophthalmology, Glaucoma Services, Fortis Memorial Research Institute, Gurgaon, Haryana, India; Associate Consultant, Department of Ophthalmology, Hong Kong Eye Hospital Chinese University of Hong Kong, Hong Kong

**Keywords:** Cataract surgery, Glaucoma, Filtering bleb, Trabeculectomy.

## Abstract

The problem of cataract management in the patients of glaucoma who have undergone fltering surgery is a challenging proposition for any surgeon, as the surgery can lead to several complications in the already compromised eye. As glaucoma requires lifelong management, the development of cataract is a significant concern because its treatment may lead to loss of intraocular pressure (IOP) control. This review aims to highlight the intra- and postoperative measures that may increase the chances of bleb survival following cataract surgery.

**How to cite this article:** Dada T, Bhartiya S, Baig NB. Cataract Surgery in Eyes with Previous Glaucoma Surgery: Pearls and Pitfalls. J Current Glau Prac 2013;7(3):99-105.

## INTRODUCTION

Data from the Advanced Glaucoma Intervention Study^1^ has shown that trabeculectomy, whether performed as primary procedure or after utilization of medical therapy, significantly increases the risk of cataract formation. All morphological types of cataract formation are increased whether it is cortical, nuclear or posterior subcapsular; with the reported increase in risk being as much as 78%. The most important determinant for the formation and rate of progression of cataract is the postoperative course following the fltering procedure. A flat anterior chamber and postoperative infammation after fltering surgery are major risk factors for cataract formation. The AGIS data has itself shown that these two complications following fltering surgery increase the risk of cataract formation by 14%.

**Figs 1A and b:**
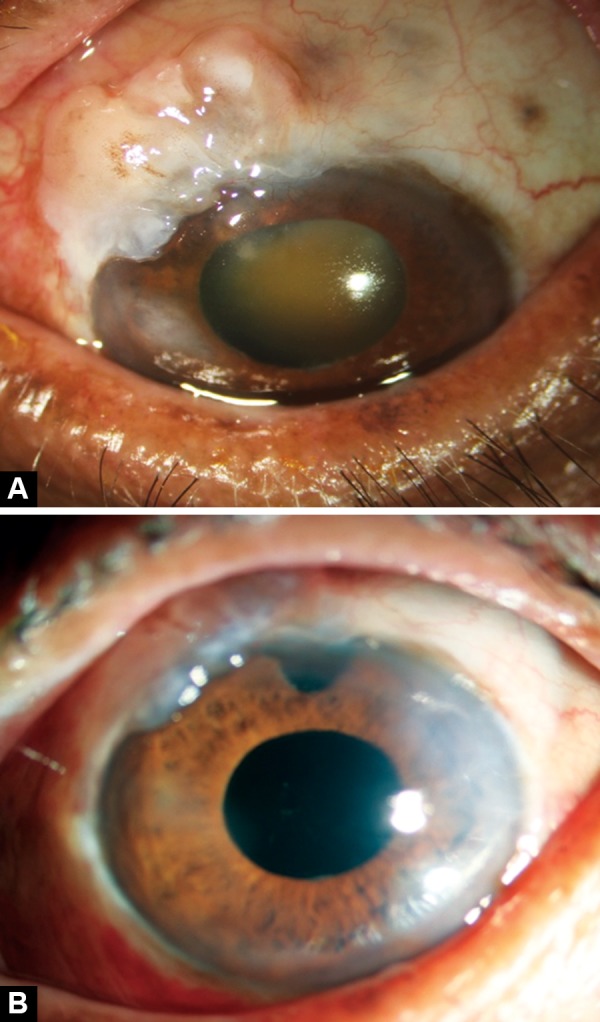
(A) Preoperative photograph showing grade 5 nuclear sclerosis (LOCS) with bleb characteristics of H_2_E_2_V_1_S_0 _(Indiana Bleb Appearance Grading System), (B) postoperative photograph showing pseudophakia with raised, functional bleb

**Figs 2A and b:**
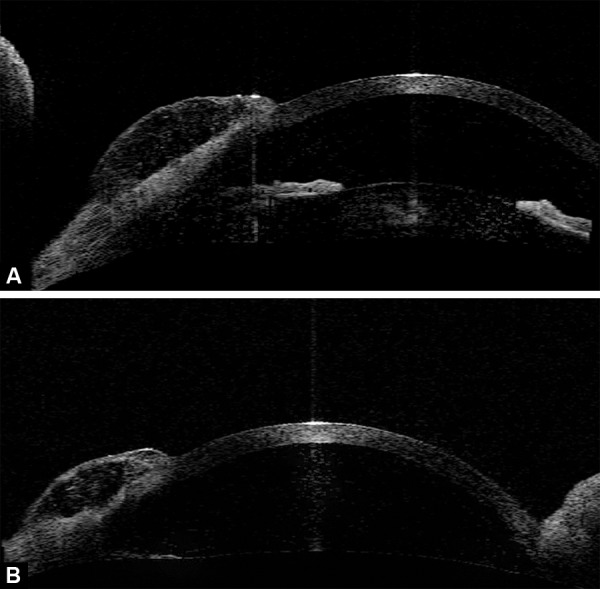
(A) Preoperative ASOCT showing a raised bleb with multiple cystic spaces suggestive of a functional bleb, (B) postoperative ASOCT showing a raised bleb with multiple cystic spaces suggestive of a functional bleb

Cataract surgery may be required at an early stage in these patients as the determination of glaucoma progression becomes increasingly difficult with the use of functional tests in the form of Humphrey visual fields. The cataract leads to declining visual field testing performance by the patient as well as their quality of life.^2^ On the other hand, cataract surgery in the patients with previous trabeculectomy is considered to have an adverse effect on long-term survival of the fltering bleb and may require special modifications (Figs 1 and 2).

### Factors that may Confound the Results of Cataract Surgery

The main objectives of the surgery are to restore optimal visual function without compromising the functioning of the bleb. The following factors need to be kept in mind when operating on eyes which may have previously undergone a trabeculectomy:

- Low corneal endothelial count
- Poor pupillary dilatation
- Floppy iris
- Shallow anterior chamber
- Hypotony
- Pre-existing zonular weakness
- Extra caution to prevent posterior capsule rupture and vitreous loss
- Postoperative uveitis
- Postoperative bleb failure
- Postoperative enhanced need for antiglaucoma medications.

### Loss of IOP Control after Cataract Surgery

The most important complication of cataract surgery in this subset of patients is the loss of intraocular pressure (IOP) control in an eye which previously had attained its target IOP. Phacoemulsification is reported to decrease the IOP as well as the need for glaucoma medication in virgin eyes. However, cataract surgery may significantly increase IOP and thereby the need for glaucoma medication in eye with pre-existing functioning fltering blebs.

Most bleb failure occurs soon after cataract surgery. Several factors are associated with an increased risk of loss of IOP control:

- Eyes with higher IOP before phacoemulsification tend to have worse postoperative IOP control and more bleb failure.
- Younger patient (aged 50 or below): advanced age is found to be good prognostic factor for control of IOP in patients undergoing cataract surgery with trabeculectomized eyes.
- Intraoperative iris manipulation.
- Early IOP spikes more than 25 mm Hg.
- Posterior capsule rupture with vitreous loss.
- Interval between trabeculectomy and cataract surgery of less than 6 months.

## PREOPERATIVE EVALUATION

It is imperative to keep in mind that the time interval between two surgeries has prognostic ramifications. If possible, it is better to delay the cataract surgery for as long as possible, without compromising the quality of life of the patient. A 6-month gap between fltering surgery and the subsequent cataract extraction increases the chances of bleb survival.

Preoperative evaluation should include a thorough clinical examination and investigations. Important aspects of the evaluation include:

- Evaluation of bleb function [slit-lamp examination/ anterior segment optical coherence tomography (ASOCT)/ultrasound biomicroscopy (UBM)].^3-5^
- IOP measurement (including diurnal control/variation, whenever possible).
- Gonioscopy to determine the patency of internal ostium of trabeculectomy.
- Extent of optic nerve damage as assessed by optic disk morphology and visual field changes.
- Corneal endothelial cell count with specular biomicroscopy.
- Corneal topography to plan placement of incision in order to minimize postoperative astigmatism.
- Assessment of pupillary dilatation.
- Evaluation of the type of cataract and lens density.
- In eyes with advanced cataract where fundus evaluation and fields are not possible, B-scan ultrasound is used to rule out gross retinal abnormalities, such as retinal detachment, and it can be used to estimate the cupping of the optic nerve head.
- Any evidence of zonular weakness and pseudoexfoliation must be looked for as such eyes require extracare in the lens manipulation during the surgery and the use of endocapsular ring for zonular support.

## INTRAOPERATIVE MEASURES

Gentle and minimal anterior chamber manipulations can prevent any inadvertent bleb trauma and minimize infammation, increasing the chances of bleb survival.

- Ideally, the cataract surgery should be performed under topical anesthesia. In case, a peribulbar block cannot be avoided, the Super pinky or Honan balloon should not be used as it can result in gross hypotony with shallowing of the anterior chamber and compression of the bleb. An intermittent massage prior to the commencement of the cataract surgery is a better option. Retrobulbar anesthesia should strictly be avoided since the risk of retrobulbar hemorrhage may compress on the compromised optic nerve leading to further visual loss or even blindness.
- Phacoemulsification in eyes with a functional fltering bleb should be performed via a clear corneal incision, away from the site of trabeculectomy. Since the site of the fltering bleb is usually superior, a temporal or slightly inferotemporal clear corneal incision ensures that the paracentesis port is also away from the bleb.
- Extra caution during paracentesis is advisable as many of these eyes have a shallow AC and there is a risk of damaging the iris or anterior capsule.
- Pupillary miosis is very commonly encountered in these cases, due to posterior synechiae, atrophy/sclerosis of the iris stroma, chronic miotic usage, previous angle closure attack, tissue aging or pseudoexfoliation. Synechiolysis, injection of high molecular weight viscoelastics or stripping of pupillary membranes may be performed when required. The pupil may then be dilated by the use of iris hooks, pupil expansion ring or other mechanical means (stretch pupilloplasty or multiple small sphincterotomies). It is important to remember that iris manipulation disrupts the blood-aqueous barrier and incites a severe infammatory reaction, jeopardizing bleb survival.
- The compromised corneal endothelium should be protected with the use of a dispersive viscoelastic, such as chondroitin sulfate or a viscoadaptive one such as 2.3% sodium hyaluronate to minimize endothelial cell loss. The use of Simmons soft shell technique also protects the corneal endothelium from further damage.
- Chilled balanced salt solution plus is the ideal irrigating fuid to use as these eyes have a low specular microscopy cell count.
- While staining the anterior capsule with dye during capsulorhexis, one can also see the staining of conjunctiva overlying the bleb through the sclerostomy ostium.^6^ This can provide an intraoperative assessment of bleb function and enable the surgeon to decide if a simultaneous internal revision of bleb is required.
- The dynamics of anterior chamber depth and bottle height in these patients is more complex than in routine phacoemulsification. These eyes tend to have shallow anterior chambers and so the height of infusion bottle should be increased to prevent collapse of the anterior chamber. The vacuum setting should also be kept on the lower side and phacoemulsification should be performed in the capsular bag by standard stop and chop or phaco-chop nucleotomy methods.
- A thorough cortical clean-up is mandatory since any retained cortical matter can excite infammation and lead to bleb failure.
- In-the-bag implantation of a square edge, single piece foldable hydroacrylic intraocular lens (IOL) is best suited for these patients. Aspheric IOLs have fast gaining popularity as the asphericity is effective in correcting spherical aberrations. Monofocal aspheric IOLs provide much better contrast sensitivity than that of multifocal IOLs. The latter, therefore, are contraindicated in glaucoma patients with significant visual field loss and attendant loss of contrast sensitivity.
- In case, there is posterior capsular rupture, sulcus fixation of a multipiece (3 pieces) hydrophobic acrylic IOL may be performed, after ensuring that the anterior chamber is completely free of vitreous strands.
- After IOL insertion, a thorough viscoelastic removal is mandatory using bimanual irrigation-aspiration handpieces. Viscoelastic must also be removed from beneath the IOL. Bleb function may be assessed by injection of BSS into anterior chamber. If the bleb is not raised following the BSS injection, an internal revision may be performed by passing a cyclodialysis spatula through the wound or paracentesis port to the sclerostomy fstula and then to the subtenon space. Gentle arcuate movements underneath the scleral fap may help to re-establish bleb function.
- In cases which are ascertained to be at risk for bleb failure, one suture may be applied to secure watertight closure of the phacoemulsification incision. Subconjunctival injection of 5-fluorouracil (5-FU) (5 mg/0.1 ml) or mitomycin C drops (0.2-0.4 mg/ml) may be considered if there is a tendency of bleb failure. Sharma et al^7^ suggested that 5-FU has a protective effect on the functioning bleb and may be used routinely at the end of phacoemulsification in such cases.

## POSTOPERATIVE CONSIDERATIONS

Close follow-up of these patients is mandatory for two reasons: firstly, to monitor the IOP which may fuctuate considerably in the immediate postoperative period; secondly, to watch out for early signs of bleb failure. The integrity of the bleb should also be monitored and if the bleb function is in question, gentle digital massage may be performed, especially in the early postoperative period.

- IOP spikes are frequently observed after cataract surgery in glaucomatous eyes in the early postoperative period, and considerable fuctuations may be noted during the first postoperative month. Therefore, the IOP must be measured regularly, and any postoperative spikes are managed using appropriate antiglaucoma medication.
- Vigorous use of topical steroids is indicated to decrease postoperative infammation, which may subsequently lead to bleb failure. However, it must be kept in mind that topical steroids may result in IOP spikes, especially in steroid responders. In such cases, topical NSAIDS have shown to be of considerable benefit, and the same may be considered as the therapeutic option of choice to minimize infammation.
- Special attention is required for the early detection of corkscrew vessels and encapsulation as signs of imminent scar formation. If there is an increase in bleb vascularization or fibrosis, subconjunctival 5-FU (5 mg) injections can be given.

**Table 1:** **Table 1:** Bleb survival following cataract surgery

*Study*		*No. of eyes (phaco/ ECCE)*		*Time between trab and cat (months)*		*Follow-up (months)*		*IOP pre-cat (mm Hg ± SD)*		*IOP post-cat (mm Hg ± SD)*		*Success (%)*		*Definition of success*	
Chen et al, 1998^8^		57/58		21.2 ± 19.1		17.6/24.5 phaco/ECCE		10.5 ± 3.9		12.1 ± 5.1		74/64 phaco/ECCE		No additional surgery or medication	
Park et al, 1997^9^		40/0		>5		>12		13.5		13.3 ± 4.1		80		IOP < 21, no additional surgery or medication	
Crichton et al, 2001^10^		69/0		Mean 2.18 years		Mean 22.0		13.58 ± 3.98		14.39		77		No additional surgery or medication	
Manoj et al, 2000^11^		21/0		>6		15.09		13.86 ± 2.94		13.25 ± 2.49		100		IOP < 21, no additional surgery or medication	
Casson et al, 2001^12^		28/0		>3		24		14.4 ± 3.5		15.3 ± 3.1		86		IOP < 21, no additional surgery or medication	
Rebolleda et al, 2002^13^		49/0		>12		19.6		12.24 ± 4.68		13.81		65.3		No additional surgery or medication	
Shingleton et al, 2003^14^		58 (26 scleral tunnel)		>4		12		11.8 ± 4.2		13.7 ± 4.6		98		IOP < 21, no additional surgery or medication	
Derbolav et al, 2002^15^		48		>3		>12		13.3		14.9		67		No additional surgery or medication	
Swamynathan et al, 2004^16^		29		>3		Mean 20.4		8.7 ± 4.5		11.8 ± 4.2		79		No additional surgery or medication	
Salaga-Pylak et al, 2013^17^		50/0		19.9 ± 12.7		18		11.6 ± 3.8		15.3 ± 3.1		71		No additional surgery or medication	

## OUTCOME OF PHACOEMULSIFICATION IN EYES WITH A FILTERING BLEB

There is considerable variability in the literature concerning the short-term and long-term effects of phacoemulsification on a functioning fltering bleb. Though the recent literature has shown no short term loss of good IOP control following cataract surgery due to improvements in surgical technique, bleb failure remains a significant concern, and increased glaucoma medications are often required, particularly in patients who need low target IOP.

Fortunately, there are now a number of studies that have attempted to address this issue (Table 1). These studies show that the IOP in patients with a functioning trabeculectomy is generally increased by up to 3.1 mm Hg after cataract surgery.^8-17^ However, the postoperative IOP is still significantly lower than that prior to the trabeculectomy. Although the definition of success varied between studies, between 65.3 and 100% of previously functioning trabeculectomies were still successful more than 1 year after cataract surgery. Rebolleda et al^13^ looked at blebs prospectively and found a decrease in the bleb in 77.6% and no bleb present in 16.3%. This is a confirmation of the general perception that there is decreased bleb function associated with cataract surgery following trabeculectomy. Halikiopoulos et al^18^ looked at bleb survival post-cataract surgery and found a significant failure rate at 3 years (30.5% requiring medication, 12.0% requiring surgery). Unfortunately, only 13 patients (33%) were operated on by clear corneal techniques.

Chen et al^8^ also identifed iris manipulation, age of less than 50 years, preoperative IOP greater than 10 mm Hg, and cataract surgery less than 6 months after trabeculectomy as risk factors for decreased bleb function after cataract extraction. These factors deserve further attention, as they may affect which patients have better prognosis for surgery and the timing of the surgery. Although it is understandable that iris manipulation could be detrimental, as it would cause breakdown of the blood-aqueous barrier and increased infammation, other studies did not find this to be a risk factor. Presumably, patients under the age of 50 would be more likely to have their trabeculectomy failed due to a more active fibrotic tendency. The finding of increased risk of failure if cataract surgery is performed within 6 months after trabeculectomy is interesting and this suggests that a bleb is more susceptible to infammation within this period of time. An IOP of more than 10 mm Hg prior to cataract surgery as a risk factor for decreased success is understandable, as patients with lower IOP are more likely to have well-established blebs and more margin to allow the IOP to rise while still staying within the target pressure zone. Interestingly, the use of MMC in the trabeculectomy prior to cataract surgery was not found to be associated with an increased chance of success. Duration of steroid use following cataract surgery may have been an important variable as well.

Crichton et al^10^ in their retrospective analysis of patients with previous trabeculectomy who underwent phacoemulsification and hydrophobic acrylic IOL implantation showed that there was a statistically increased number of medications and IOP of patients postoperatively. The maximum increase was noted at the 1 year follow-up visit. Similar results were seen in the study published by Yamagami et al^19^ in which the pre-existing fltering bleb was greatly reduced in size in 56% of eyes after phacoemulsification at 2 years and the level of IOP control was worsened in 66% of the eyes in which the fltering bleb size was greatly reduced.

On the other hand, Liu et al^20^ in their study concluded that glaucomatous eyes with cataract after a filtering operation, can rehabilitate their visual acuity and maintain the functional fltering bleb by phacoemulsification with foldable IOL implantation through a temporal clear corneal incision or lateral scleral pocket incision. No IOP changes have been reported after phacoemulsification (temporal clear corneal incision) in trabeculectomized eyes.

Casson RJ et al^21^ compared the effect of phacoemul-sification with the IOL implantation on long-term IOP control in glaucoma patients with the effect on IOP control after extracapsular cataract extraction (ECCE) with IOL implantation and concluded that phacoemulsification provided better long-term IOP control than ECCE.

Investigators have studied outcomes in patients with existing, well-functioning blebs who underwent phacoemulsification and found that modern cataract surgery, whether performed through a temporal clear corneal incision or temporally via a scleral tunnel, restores visual acuity without jeopardizing bleb function. No statistically significant differences were found in postoperative visual outcomes and IOP.

The AGIS investigators reported the incidence and outcomes of cataract on visual fled indices in a separate report.^1^ A total of 47.2% of the 778 enrolled patients in the AGIS developed cataract. The follow-up period was 7 years. They concluded that a more significant improvement in visual acuity occurs following cataract surgery than visual field. On the visual fields, only the mean deviation tends to improve whereas measures of localized field depression, such as pattern deviation and corrected pattern standard deviation, remain unchanged or even increase indicating glaucoma progression.

Cataract surgery has also been advocated to treat postfiltering surgery hypotony maculopathy and chronic choroidal detachment in those patients with coexisting cataract. Sibayan et al^22^ reported that routine phacoemulsification surgery resulted in the resolution of hypotony and visual complaints in patients with postfltering hypotony. In a similar study, Doyele et al^23^ tried modifed technique of phacoemulsification leaving viscoelastic in eyes at case conclusion. The author observed that this helped in decreasing initial aqueous fow through the bleb and, thereafter, minimizing topical steroid use results in an increased infammation and the associated decrease in bleb size and function to resolve hypotony after trabeculectomy.

There are very few studies on phacoemulsification in operated glaucoma valves. Gujral et al^24^ retrospectively reviewed records of 19 patients (23 eyes) who had a phacoemulsification more than 3 months after insertion of an Ahmed glaucoma valve (AGV). Four eyes had an IOP increase >10 mm Hg on day one. The mean IOP or number of medications did not significantly change after phacoemulsification at 1 month or thereafter (p > 0.05). However, one eye required a second AGV.

On the contrary, Patel et al^25^ concluded in their review that subsequent cataract surgery can affect IOP control following trabeculectomy but not after tube shunt surgery. Measures, such as intraoperative subconjunctival 5-FU injection and repeated postoperative 5-FU injection may minimize the risk of bleb failure and loss of IOP control in high-risk patients.

In addition to bleb function, Muallem et al^26^ has demonstrated in their retrospective, interventional, case control study that lower prephacoemulsification IOP was weakly correlated with a myopic shift in the final refraction as compared to the predicted refraction in patients with phacoemulsification (p = 0.017). This can be a useful reference but a prospective study of a larger sample size is warranted.

## CONCLUSION

The wide variety in the results from various parts of the world lead us to the conclusion that taking decision about cataract surgery in an operated trabeculectomy patient is a very tricky situation and utmost care should be taken to avoid potential postoperative complication. The most important of which is the failure of the existing bleb. Due care given to each step as mentioned above can give good results with no detrimental changes in postoperative IOP and bleb status as evident from the published literatures. Patients should be maintained on long-term follow-up with regular check-up of IOP and visual fields, and to detect any change in the bleb morphology as well as functional deterioration of the optic nerve.

## KEYPOINTS

1. Delay cataract surgery until the bleb has matured (at least 6 months).
2. Assess bleb function before surgery.
3. Evaluate pupillary dilatation.
4. Minimize conjunctival trauma.
5. Utilize a small clear corneal incision far from the bleb (temporal if possible).
6. Assess bleb function upon completion of the cataract surgery and revise if needed.
7. Aggressively treat postoperative infammation and IOP spikes.
